# Neuronal Migration During Development of the Cerebellum

**DOI:** 10.3389/fncel.2018.00484

**Published:** 2018-12-17

**Authors:** Maryam Rahimi-Balaei, Hugo Bergen, Jiming Kong, Hassan Marzban

**Affiliations:** ^1^Department of Human Anatomy and Cell Science, Max Rady College of Medicine, Rady Faculty of Health Sciences, University of Manitoba, Winnipeg, MB, Canada; ^2^The Children’s Hospital Research Institute of Manitoba (CHRIM), Max Rady College of Medicine, Rady Faculty of Health Sciences, University of Manitoba, Winnipeg, MB, Canada

**Keywords:** neuron, migration, cerebellum, development, mechanism

## Abstract

Neuronal migration is a fundamental process in central nervous system (CNS) development. The assembly of functioning neuronal circuits relies on neuronal migration occurring in the appropriate spatio-temporal pattern. A defect in the neuronal migration may result in a neurological disorder. The cerebellum, as a part of the CNS, plays a pivotal role in motor coordination and non-motor functions such as emotion, cognition and language. The excitatory and inhibitory neurons within the cerebellum originate from different distinct germinal zones and migrate through complex routes to assemble in a well-defined neuronal organization in the cerebellar cortex and nuclei. In this review article, the neuronal migration modes and pathways from germinal zones to the final position in the cerebellar cortex and nuclei will be described. The cellular and molecular mechanisms involved in cerebellar neuronal migration during development will also be reviewed. Finally, some diseases and animal models associated with defects in neuronal migration will be presented.

## Introduction

The structural and functional development of the central nervous system (CNS) depends on neurogenesis, neuronal migration and circuit formation. This is a complex sequence of events involving a variety of molecular pathways. Neuronal migration is an essential phenomenon for normal development as it brings cells into appropriate spatial relationships with other cells (Marín et al., 2010). During development, newborn neurons form within the neuroepithelium, a proliferative layer of the neural tube. Under tightly controlled conditions, the newborn neurons migrate from their germinal zone and disperse throughout the CNS to reach their final destination where they subsequently become part of an appropriate lamination and neuronal circuit (Cooper, 2013). Cell polarity is required for neuronal migration which is dependent on cytoskeletal changes in concert with cell adhesion receptor systems that are regulated by a wide variety of molecules (Govek et al., 2011). Neuronal locomotion can be divided to three phases: (1) extension of the leading process; (2) nucleokinesis; and (3) retraction of the trailing process (Tsai and Gleeson, 2005). The main mode of neuronal migration is somal translocation which uses extracellular matrix components (Nadarajah and Parnavelas, 2002), glial fibers (Hatten, 1990), blood vessels (Tsai et al., 2016), axons (Takei et al., 2000) and possibly pia mater (Komuro and Yacubova, 2003) as substrates. Neuronal migration follows either a radial or a tangential migratory pathway, depending on the area of the developing nervous system in which the neurons originate. In radial migration, nascent neurons follow a track that is perpendicular to the neuroepithelial surface and the neurons proceed alongside radial glial fibers (Marín and Rubenstein, 2001). In contrast, the tangential migration of neurons is parallel to the pial surface (Nadarajah et al., 2001). There is also a dual phase neuronal migration referred to as a switching migration, which is a combination of tangential and radial migration (Kawaji et al., 2004).

In this article, the neuronal migration modes and the direction and pathways from origin to the final position during CNS development will be reviewed. Then, we will review the migration of cerebellar neurons with an emphasis on Purkinje cells (PCs). Finally, we will describe some diseases and animal models associated with defects in neuronal migration.

## The Cellular and Molecular Mechanisms Involved in Neuronal Migration

During neuronal migration, neuronal precursor cells move forward, switch their direction, or change their migration mode to reach their final position, which is fundamental for nervous system function. All of these processes are under an elaborate control system and have been studied extensively (Table 1). In this section, we will describe the main molecular and cellular mechanisms involved in neuronal migration during CNS development and then the migration of each cerebellar neuronal type will be described.

**Table 1:** Methods to study neuronal migration (Mannan et al., 2004; Baubet et al., 2012; Rahimi-Balaei et al., 2016).

Techniques for progenitor differentiation and neuronal migration	
Traditional	a) Analysis of fixed tissue b) *in vitro*: culture of individual cells
Advanced	a) Electron microscopy b) Tissue culture methods c) Chimeras d) X-ray crystallography e) Genetic labeling
Recent	a) Live imaging techniques b) Genetic labeling of distinct cell types in developing brain c) *In vitro* migration assay using dissociated neuronal cells migration (boyden chamber assays and gap closure assays) d) Real-time neuronal migration in embryonic brain slice assay (fluorescent dyes or XFP transgenes, lipophilic or vital dyes, such as DiI, DiO, CMTMR, Oregon Green plus dye- or transgene-coated gold particles *in utero* or *ex vivo* electroporation) e) Neuronal migration in embryonic brain explants in 3-D matrigel f) Embryonic culture g) Dynamic *in-silico* model for neuronal migration

Migrating neurons exhibit highly polarized cell morphology in the direction of their movement. The polarized neurons are defined as having a leading process and a trailing process. The leading process is a structure that is similar to the growth cones of growing axons, whereas the trailing process is a short process at the posterior part of the cell. The formation of these processes is regulated by precise cellular and molecular mechanisms through which extrinsic and intrinsic signaling pathways change the cytoskeleton resulting in pulling and pushing forces (Matsuki et al., 2013; Nguyen and Hippenmeyer, 2013). The major structures that define the leading edge activity of migrating neurons are lamellipodia and filopodia (Kurosaka and Kashina, 2008). Initially a lamellipodium-like network forms and then filopodia form through the addition of monomers to filaments and assembly with adjacent filaments (Davies, 2013). Lamellipodia are broad membrane protrusions at the leading edge of cells that arise as a result of actin polymerization. Lamellipodia are dynamic structures that include protrusion and retraction activities (Krause and Gautreau, 2014). On the other hand, filopodia are thin protrusions of the lamellipodium plasma-membrane. The formation of filopodia is a highly dynamic process and these structures function as antennae to navigate and direct cell migration. The initiation and elongation of filopodia depends on the precise regulation of polymerization, crosslinking and assembly by various actin-associated proteins (Mattila and Lappalainen, 2008).

The movements of neurons are controlled by the generation, maintenance and remodeling of a leading process. The leading process of the neuron marks the direction of neuronal migration, followed by movement of the cell somata (somal translocation) along with the translocation of the nucleus (nucleokinesis), and finally the migrating neuron eliminates its trailing process. Leading processes interact with the surrounding microenvironment to guide neuronal movements (Nguyen and Hippenmeyer, 2013). The remodeling of the leading process will repeatedly initiate new migratory cycles until it reaches its final destination (Nguyen and Hippenmeyer, 2013). Cytoskeletal proteins such as microtubules, actin and actomyosin play important roles in nucleokinesis and cell locomotion. The centrosome is the main microtubule organizing center and as it moves forward, it pulls forward the longitudinal array of microtubules in association with the Golgi apparatus, which is followed by the movement of the nucleus. The absence of microtubules at the trailing part of the cell may initiate contractions dependent on myosin II, and this pushing force on the nucleus results in moving forward and breaks adhesions at the trailing part of the cell. The role of actomyosin contraction at the back part of the cell also plays an important role in the migration of cortical interneurons (INs; Martini and Valdeolmillos, 2010). The somal translocation process is the main mode of neuronal migration during the early stage of embryonic development and includes the radially migrating neurons such as cerebellar granule cells (GCs) that move along the Bergmann glia fibers. A wide range of cellular events, including cell adhesion, modulate this migration (Hatten, 1999; Nadarajah et al., 2001; Sanada et al., 2004).

It has been shown that Lissencephaly-1 homolog, (LIS1, a member of the microtubule-associated proteins, MAPs) and doublecortin (DCX, a member of MAP that directly polymerizes purified tubulin into microtubules) are important in the translocation of the neuronal cell body during neuronal migration. Both molecules are components of an evolutionarily conserved pathway regulating microtubule function and cell migration (Gleeson et al., 1999; Feng and Walsh, 2001). In addition, the microtubule bundling that is accompanied by the action of dynein mediates coupling of the nucleus to the centrosome (modulating and stabilizing microtubules; Tanaka et al., 2004). In another study, it has been shown that LIS1 and dynein play a role in radial neuronal migration (Wynshaw-Boris and Gambello, 2001). In males, DCX mutations produce lissencephaly phenotypes similar to those associated with *LIS1* mutations (Gleeson et al., 1998). Recently, c-Jun N-terminal signaling pathway has gained attention as one of the critical regulators of neuronal mobility. Indeed, components of this pathway activate some specific brain proteins (e.g., by phosphorylation of the MAP1B and MAP2) which affect the stability of microtubules in neurons and neuronal migration (Tsai et al., 2016). In cerebellar GC migration, that is assisted by Bergmann glia, the majority of F-actin and myosin II is located at the front of the nucleus rather than the trailing end, suggesting these proteins may pull the soma forward (Solecki et al., 2009).

In addition to intrinsic factors discussed above, there are several extrinsic factors (also known as motogens) involved in neuronal migration. Gamma amino butyric acid (GABA) secreted by neurons themselves, acts as an extrinsic factor and accelerates their migration. In mice deficient in GABA, the migration rate of neurons is decreased, which is consistent with a role for GABA as a motogen (Inada et al., 2011). Hepatocyte growth factor/scatter factor (HGF/SF) is another extrinsic factor involved in migration. In mice lacking the urokinase-type plasminogen activator receptor (uPAR, a key component of HGF/SF activation), neurons exhibit abnormal migration from the ganglionic eminence, which leads to a reduced number of neurons in the frontal and parietal cortices (Powell et al., 2001).

The rate of cerebellar GC migration is controlled positively through the frequency of the intracellular calcium fluctuation and negatively regulates the rate of the extension of axonal growth cones. In cortical migratory interneurons, their motility is stimulated by the activation of GABA and glutamate receptors. An up-regulation of the potassium-chloride co-transporter (KCC2) plays a key role in reducing interneuron motility through its ability to reduce membrane potential upon gamma-aminobutyric acid A(GABA_A_) receptor activation, and decrease the frequency of intracellular calcium transients. Subsequently, during early postnatal weeks the expression of KCC2 is increased and early-born interneurons express higher levels of KCC2 compared to late-born interneurons (Bortone and Polleux, 2009).

The control of the specific direction of the migration in cortical neurons originating from the subventricular zone (SVZ) is a combination of the leading process and the use of scaffolds (such as the radial glia; Marín et al., 2010). However, there are additional factors such as semaphorin 3A (Sema3A), which acts like an attractant and is expressed in descending gradients across cortical layers, that guide newborn cortical neurons to the upper cortical layers (Chen et al., 2008). During development of pontine nuclei in the hindbrain, neurons reach the midline and Netrin-1 acts as a midline attractant and these neurons themselves express deleted in colorectal cancer (DCC), a Netrin-1 receptor), to assist these neurons to reach the midline (Yee et al., 1999). Neuregulin-1 (NRG1) is a member of the NRG family of proteins that contains an epidermal growth factor (EGF)-like motif that activates EGF receptor. It is expressed in the developing cortex and acts as a chemoattractant. Erb-B2 Receptor Tyrosine Kinase 4 (ErbB4), the NRG1 receptor, is expressed in migrating interneurons (Flames et al., 2004).

It is clear that several intrinsic and extrinsic factors are involved in the regulating neuronal migration. The mode of the neuronal migration, its direction, and finally the positioning of the neurons, which is important for neuronal circuit formation and function, are regulated by a complex molecular pathway that is currently not fully understood and need to be addressed in future. In the next section, we discuss direction of neuronal migration. The orientation and directionality of cell migration can be classified into two basic axes (radial and tangential) that use different types of substrates such as glial processes or neuronal axons (Rakic, 1990).

### Radial Migration

Radial migration occurs in two opposite directions: (1) pial-directed migration in which neurons migrate toward the pial surface (or the outer neural tube surface); and (2) radially inward migration in which neurons migrate away from the pial surface. In the pial-directed radial migration, the neural progenitors or neuroepithelial derived cells migrate from their site of origin toward the pial surface to reach the mantle zone. Radial glial cells, which express glial fibrillary acidic protein (GFAP), play an important role in promoting the generation of neuronal progenitors and providing the migratory substrate during the neuronal migration (Tabata and Nakajima, 2003). Although pyramidal cells of the cerebral cortex are the classical example of pial-directed radial migration, a recent study has demonstrated this movement is not as straight forward as previously thought. Recently, it has been described that neurons may switch the mode of their migratory pathway (in the intermediate and subventricular zone, IZ/SVZ) before starting radial migration (Tabata and Nakajima, 2003).

During inward radial migration, neurons move away from the pial layer after their tangential migration. The typical examples for this mode of migration are cerebellar GCs and pontine nuclei neurons (Kawauchi et al., 2006).

### Tangential Migration

Tangential migration occurs in two different manners: (1) directed; and (2) non-directed. In the directed manner of migration, many neurons and interneurons migrate tangentially from their site of origin toward a specific direction. This includes cerebellar granule cell precursors (GCPs), interneurons of the cerebral cortex, neurons of the pontine nuclei, Cajal Retzius cells, neurons of the lateral reticular nucleus, and neuronal migration from the telencephalon to the olfactory bulb. The interneurons of the cerebral cortex migrate between the pallium and subpallium and the neurons of the pontine nuclei migrate between rhombomeres (Nóbrega-Pereira and Marin, 2009). Cajal-Retzius cells play an important role during neuronal migration as they secrete Reelin to guide the radial migration of the projection neurons of the neocortex. These cells originate from the discrete pallium and by tangential migration they will colonize the surface of the entire cortex (Bielle et al., 2005; Gil-Sanz et al., 2013).

The non-directed manner of tangential migration is more complicated than other modes of the migration, in that some groups of neurons exhibit migrations in all directions of the tangential plane. For example, interneurons of the marginal zone (MZ) of the cerebral cortex migrate tangentially in different directions, or change their direction repeatedly, which is referred to as random walk (Tanaka et al., 2009).

### Switching Migration (Mode and Direction)

Although many neurons migrate simply to reach their final destinations, for some neurons the migration is more complicated and involves a type of migration referred to as switching migration (random walk). Switching migration can occur dynamically and includes switching from radial to tangential migration or directed to non-directed manner, and vice versa. For example, tangentially migrating cerebellar GCPs in the external germinal zone place a leading and a trailing process oriented horizontally and then orient these processes vertically to the putative molecular layer from the cell body (Komuro and Yacubova, 2003). After a stationary period following the tangential migration, the GCs switch to radial migration and migrate to the direction of their descending processes (Komuro and Yacubova, 2003). Similarly, pontine nuclei neurons also switch their mode of migration from tangential to radial as they approach the region of the pontine nuclei. The leading processes of pontine nuclei neurons divert their direction radially and start radial migration. In some neurons of the pontine nuclei, the new-born process initially elongates radially and subsequently results in the radial migration of their soma (Hatten, 1990).

The basic mechanisms and principles of neuronal migration during development that are described above are general and similar for most of the neuronal types in the CNS. However, depending on the area of the developing CNS, diverse classes of neurons follow different strategies and may use distinct molecular cues and substrate during migration from their origin to their final position. Based on this, the following sections will focus on neuronal migration in the cerebellum.

## The Neuronal Migration in the Developing Cerebellum

Similar to the other regions of the brain, neuronal migration plays a substantial role in the development of cerebellar circuits (Hoshino et al., 2005). Relatively few cell types are aggregated to form the cerebellar gray matter, which includes the cerebellar cortex and the cerebellar nuclei (CN). The neurons that reside within the cerebellum are derived from two distinct germinal zones: the VZ and the rostral rhombic lip (RL). The VZ is the neuroepithelium of the alar plate of rhombomere 1 that will form the roof of the 4th ventricle. The neurons derived from the VZ includes PCs, Golgi cells, stellate cells and basket cells (Butts et al., 2014). All of these neurons are derived from neural progenitors that express pancreas specific transcription factor 1a (Ptf1a) and use GABA as a neurotransmitter (Hoshino et al., 2005). The cerebellar neurons derived from the RL at the dorsal edge of the cerebellar primordium, include the large neurons of the CN (which provide the output of the cerebellum), unipolar brush cells (UBCs), and the GCs (the most numerous cell in the brain; Elsen et al., 2013). All of these neurons originate from neuronal progenitors that express Atonal homolog 1 (Atoh1, formerly known as Math1) and use glutamate as their neurotransmitter (Manto et al., 2013).

The cerebellar cortex segregates into three layers: the molecular layer (stellate and basket cells), the PC layer (PCs and candelabrum cells) and the granular cell layer (GCs, Golgi cells, UBCs and Lugaro cells). The two most distinctive cells in the cerebellar cortex are the large PCs and the small GCs. PCs are the principal neurons of the cerebellar cortex, and the sole output of the cerebellar cortex projecting an axon to the CN. The molecular layer of the cerebellar cortex contains inhibitory interneurons, but is dominated by PC dendrites and parallel fibers which are the axons of GCs (Butts et al., 2014). PCs develop earlier and initially secrete sonic hedgehog (Shh) which is essential for proliferation of GCPs (Wallace, 1999). Under an intricate regulatory system, the appropriate numbers, migration and positioning of these cells is required in order for synapse formation and assembly of the cerebellar cortical circuitry.

The cerebellar cortex, which is the location of the most of cerebellar neurons, is compartmentalized and the cytoarichtecture is the most elaborately patterned circuit of all the CNS structures (White and Sillitoe, 2013; Beckinghausen and Sillitoe, 2019). The molecular expression patterns, afferent/efferent fibers, and birthdates divide the cerebellar cortex into an array of parasagittal stripes (e.g., Voogd, 1967; Hashimoto and Mikoshiba, 2003; Sugihara and Shinoda, 2004; Pijpers et al., 2006; Apps and Hawkes, 2009; Marzban and Hawkes, 2011; Bailey et al., 2013; Rahimi-Balaei et al., 2016) and is further subdivided into four transverse zones (Sillitoe et al., 2005; Marzban et al., 2008; Marzban and Hawkes, 2011; Bailey et al., 2013, 2014; Rahimi-Balaei et al., 2016). The most extensive study of cerebellar cortex compartmentation was performed on PCs using zebrin II and phospholipase C beta 4, and resulted in a striking map of topographic stripes (Plcβ4) e.g., (Marzban et al., 2007; Kim et al., 2009; Bailey et al., 2014). The cerebellar cortical interneurons are also organized and restricted to the same zone and stripes pattern (Consalez and Hawkes, 2013). In addition, stripes of the cerebellar cortex align with the terminal fields of the two major cerebellar afferent types; mossy fibers and climbing fibers (Akintunde and Eisenman, 1994; Sugihara and Shinoda, 2004; Sugihara and Quy, 2007; Rahimi-Balaei et al., 2015; Sillitoe, 2016). Remarkably, an interesting birth dating study has revealed that the zone and stripe pattern is established before migration of cerebellar neurons. It was found that, the fate of PC topography is already specified according to their birth date during E10.5–12.5 (Hashimoto and Mikoshiba, 2003). Recently, it has been shown that the CN neurons (CNNs) are organized with molecular heterogeneity that may mirror the molecular complexity of the cerebellar cortex (Sugihara and Shinoda, 2007; Chung et al., 2009a; Sugihara, 2011).

**Figure 1:**
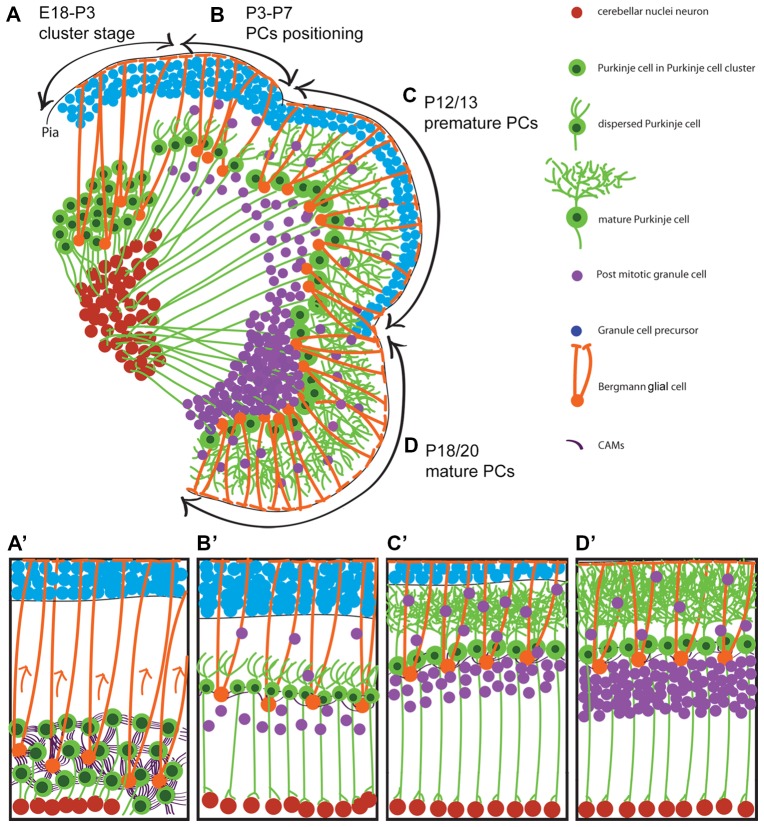
Neuronal migration during postnatal cerebellar development. A schematic illustration of cerebellar development at sagittal section of postnatal mice. It shows that Purkinje cells (PCs) cluster disperse to establish PC monolayer and start maturation while granular layer form from external germinal zone (E18–P20). **(A,A′)** Around E18 to P3, PCs are in clusteric stage and show high CAMs connections between PCs-PCs and PCs-Bergmann glial cell body which is located beside PCs during clusteric stage and extend their fibers to the cerebellar pia surface. **(B,B′)** PCs in dispersal situation and positioning process (P3–P7) with less CAMs connections and show shortened Bergmann glial fibers. **(C,C′)** It shows how premature PCs (P12/13) arborized (maturation process) while granule cell precursors (GCPs) migrate to the developing granular layer and become mature GCs. **(D,D′)** At around P18/20 is end stage of the PCs maturation and GCPs inward migration. P, postnatal day; E, embryonic day; CAMs, cell adhesion molecules.

Underlying the complex cerebellar cytoarchitecture with a few neuronal types is an intricate sequence of events in which neurons that originate from different germinal zones migrate via a complicated migratory pathway to their final position and establish elaborate cerebellar compartmentation and circuits (Figure 1). The cerebellar glial cells migration are not included in this review because of complexity and the molecular processes involved and need to be discussed in a specific focused review article.

### Purkinje Cells Origin, Migration and Final Organization

PCs, which are key neurons in the cerebellar cortex networking, complete their final mitotic division at E10.5–12.5 in mice (Hashimoto and Mikoshiba, 2003, 2004; Minaki et al., 2008). Once in the postmitotic stage, they start a short distance radial migration along the radial glial fibers (scaffold/substrate) from their site of origin in the VZ (Rakic and Sidman, 1970; Yuasa et al., 1996; Hatten, 1999). PCs exhibit an elongated morphology consistent with axonogenesis at E12.5, which is directed toward the mantle zone where they accumulate as an irregular multilayer of cells called the cerebellar plate, or PC plate (PCP) at E14.5 (Miyata et al., 2010). However, PC axons project to the CN by around E16 (in mice; Sillitoe et al., 2009) and around E18 (in rat; Eisenman et al., 1991). During the early stage of PC migration, PCs initially have a leading apical neurite and trailing process, and the cell’s position changes during cerebellar primordium expansion and morphological development (Hatten and Heintz, 1995; Sotelo and Dusart, 2009). At E13, the somata of cerebellar radial glial cells—the precursor of Bergmann glia—are aligned along the surface of the 4th ventricle, and extend processes up to the pial surface of the cerebellar plate. Along with translocation of the radial glial somata from VZ, the PCs undergo delamination and migration which is quickly followed by the detachment of the trailing process of the radial glia from the VZ (Yuasa et al., 1996). The migration of PCs along radial glia has not been documented in live preparations and this assumption is based on still images. However, it is believed that the newborn Purkinje cells from the VZ migrate radially, along the processes of radial glia. Recently it was shown that those Purkinje cells originate from caudolateral VZ migrate tangentially and cross the radial glial fibers but not along with them (Miyata et al., 2010; Sotelo, 2017; Schilling, 2018). With this evidence it can be speculated that despite the tangentially oriented PCs, some of these cells use radial glial fibers for their migration. The fast proliferating VZ neuroepithelium leads to prominent bulging toward the 4th ventricle and the caudal appearance of the cerebellar primordium seems oriented horizontally. However, radial glial fibers are connected from the neuroepithelium to the subpial surface and provide substrates for the PCs that originated from the caudal VZ.

After ~E14.5 the PCP, a multi-cell-thick immature PC layer, is expanded in orchestra with the cerebellum development and aggregated in several PC clusters (PCCs) that become well established around E17.5 (Fujita et al., 2012). It appears that there is no migratory activity during this stage, but rather a small displacement of Purkinje cell groups following expansion of the developing cerebellum. The second wave of PC migration/displacement is initiated after formation of the PCCs. PCs disperse and arrange in a single regularly spaced monolayer during cerebellar expansion and simultaneously grow their axon and dendrites (Butts et al., 2014). During PC differentiation, they collapse their apical neurite (at P0) and form numerous short neurites at ~P6 which develop ramified dendrites at ~P8 (Armengol and Sotelo, 1991). In humans, PC progenitors start their migration from the VZ at stages18 and 19 of the development (~44–48 days; Müller and O’Rahilly, 1990). They form a monolayer at 16–28 weeks of the gestation, and start the cerebellar enlargement with the development of more dendritic branches, which is associated with an increase in synapse formation (Müller and O’Rahilly, 1990).

It is not clear how PCs disperse from the cluster stage to the monolayer position (Figure 1). One of the most studied molecules that is involved in controlling PC migration is the Reelin pathway (Larouche and Hawkes, 2006). During mouse cerebellar development, the expression of Reelin (*Reln* mRNA and glycoprotein) is first detected at E13, along the dorsal cerebellar surface corresponding to the RL-derived cells and the nuclear transitory zone (NTZ; Fink et al., 2006). It has been shown that the delamination of postmitotic lateral Purkinje cells (at around E10.5) from the ventricular neuroepithelium and their initial migration is independent on Reelin signaling because at this time-point Reelin expression in the cerebellum has not yet started (Yuasa et al., 1993). By the first week of postnatal life, Reelin expression disappears from the deep areas but is maintained in GCPs, and the expression of Reelin may continue in some GCs of adult mice (Fink et al., 2006). Dispersal of Purkinje cells from the clusteric stage is dependent on Reelin expression and its downstream molecules apolipoprotein E receptor 2 (ApoER2) and very-low-density lipoprotein receptor (VLDLR). Reelin binds with similar affinity to ApoER2 and VLDLR. Disabled-1 (Dab1) which is a key molecule in the Reelin pathway, is expressed by Purkinje cells, as they settle underneath the Reelin-expressing cells of the external germinal zone (Fatemi, 2005; Miyata et al., 2010). Mutations in the *Reelin* gene (*reeler* mouse; D’Arcangelo et al., 1995), *dab1* gene (*scrambler* and *yotari* mouse; Howell et al., 1997; Sheldon et al., 1997), and targeted deletion of the genes for both *Apoer2* and *Vldlr* (Trommsdorff et al., 1999) all result in a similar phenotype of ectopic Purkinje cells due to a lack of dispersal from the cluster. These findings have placed Reelin, ApoER2, VLDLR and Dab1 into a common signaling pathway. It has been shown that the Reelin protein resembles extracellular matrix proteins that are involved in cell adhesion (D’Arcangelo et al., 1995) and regulate cadherin function via Dab1.

Cadherins are a group of transmembrane proteins that mediate cell–cell adhesion during tissue morphogenesis (Maitre and Heisenberg, 2013). Cadherin-6B (also known as cadherin-7) is overexpressed in Purkinje cell progenitors and is involved in guiding migrating neurons along neurites that express the same cadherin during their radial migration. These findings indicate that cadherin adhesive mechanisms are involved in neuronal guidance (Takei et al., 2000). It has also been shown that Dab1 signaling controls the adhesive property of neurons to radial glia. The newborn neurons in the cortex of *scrambler* mice remain attached to the process of their parental radial glia during the entire course of radial migration and this abnormal neuronal-glial adhesion is highly linked to the positional abnormality of neurons in *scrambler* mice. Additionally, the phosphorylation of tyrosine residues of Dab1 regulates α3 integrin levels in migrating neurons and their timely detachment from the radial glial fibers (Sanada et al., 2004). Furthermore, direct interaction of phosphorylated Dab1 with other intracellular proteins such as members of Crk (adapter molecule crk also known as proto-oncogene c-Crk or p38) family might connect the Reelin pathway to integrin-mediated adhesion and migration of neurons (Ballif et al., 2004; Mayer et al., 2006). It has been shown that abnormal migration of neurons in *scrambler* mice is associated with an impaired detachment of neurons from clonally related radial glial cells. This abnormal neuronal-glial adhesion depends on α3 integrin signaling that is regulated by Dab1 (Sanada et al., 2004). However, a major challenge still remains: how do Purkinje cells from the clusteric stage disperse to their position in a monolayer?

It has been suggested that the PCC position changes during cerebellar surface development, which is extended rostrocaudally and mediolaterally and becomes arranged in a monolayer due to cerebellar expansion (Butts et al., 2014). In addition, GCs have been proposed to be a major player in the positioning of Purkinje cells postnatally (Jensen et al., 2002). However, several reports have shown that, Purkinje cells respond differently to different GC defects (such as hypoplasia, agenesis). In most cases, each affected cerebellum comprises several small percentages of Purkinje cells population, which are either in different ectopic locations, or arranged in the monolayer position. For example, a study of the *math1* null-mutant mouse in which the external germinal zone does not form, three populations of ectopic Purkinje cells have been described (Jensen et al., 2002). In the *scrambler* (mutation in *Dab1*, Reelin adaptor protein), the cerebellum is small because the size of the GC population is severely diminished by ~80% and approximately 95% of Purkinje cells (not all) fail to complete their migration (Goldowitz et al., 1997; Reeber et al., 2013). Chemokine receptor 4 (*Cxcr4*) deficiency results in a lower number of GCs in the cerebellum and partially disorganized ectopic Purkinje cells (Huang et al., 2014), which is very similar to the phenotype described in *Weaver* mice (Smeyne and Goldowitz, 1989; Chen et al., 2009). It has been suggested that protein tyrosine phosphatase, non-receptor type 11 (Ptpn11) regulates formation of the laminar cerebellar cortex by controlling GC migration via the Cxcl12/Cxcr4 signaling (Hagihara et al., 2009), although removing *Ptpn11* in the external germinal zone has no distinct effect on cerebellar corticogenesis (Li et al., 2014). These results indicate that GCs are not the main player driving Purkinje cell organization in the cerebellum. Such an elaborate Purkinje cell monolayer organization cannot be explained by surface expansion and GC development. The development of this monolayer organization must be precisely regulated by active cellular and molecular processes rather than by a passive expansion.

Do PCs use any substrates or cells such as Bergmann glia cells to disperse from the clusteric stage to their final destination? Sudarov and Joyner (2007) introduced the role of GCs and Bergmann glia during formation of the base of each fissure (as an anchoring center) and proposed that this dictates the shape of the folia. Bergmann glia are defined as PC-associated astrocytes and are a specific type of astrocyte, which are zonally organized in the cerebellar cortex (Reeber et al., 2018). Bergmann glial cells originate from radial glia within the VZ. The radial glia transform to Bergmann glial cells during E14.5–E18.5 (in mice) under control of Ptpn11, which maintains the basal processes of the radial glia and relocates somata from the VZ to the nascent PCCs (Yuasa, 1996; Li et al., 2014). It is well documented that Bergmann fibers are associated with GCs in migration during cerebellar postnatal development, and this is the origin of the concept of glia-guided neuronal migration (Hatten, 1990). Recently, it was suggested that Bergmann glial cells are essential in cerebellar corticogenesis, especially through monolayer formation of Purkinje cells, dendritogenesis, migration of GCs, and circuit formation (Cajal, 1911; Rakic, 1971; Altman and Bayer, 1997; De Zeeuw and Hoogland, 2015; Leung and Li, 2018). Yamada et al. (2000) showed that not only are Bergmann glial cells associated with Purkinje cells in the adult cerebellum but they are also associated with Purkinje cells during their migration, dendritogenesis, synaptogenesis and maturation (Yamada and Watanabe, 2002). It is still controversial whether Bergmann glia regulate Purkinje cell monolayer formation through Notch-RBP-J signaling and notch ligand, Delta-like 1 (Komine et al., 2007; Hiraoka et al., 2013), since ablation of genes from Bergmann glia does not affect Purkinje cell monolayer formation while ablation of *Dner* (delta/notch-like EGF receptor containing) from Purkinje cells results in Bergman glia disruption (Eiraku et al., 2005; Tohgo et al., 2006; Greene et al., 2016). Bergman glia are rich in glutamate receptors and transporters [SLC1A3 (GLASTs) or the excitatory amino acid transporter, EAAT1] that are involved in Purkinje cell synapse formation (O’Hearn and Molliver, 1997). In fetal and neonatal stages, SLC1A3 is expressed ubiquitously in cerebellar radial glia or astrocytes and excessively in Bergmann glia at the postnatal stage (Yamada et al., 2000). It has been shown that the dendrites of the growing Purkinje cells ascend through the GFAP^+^/SLC1A3^+^ rod—like Bergmann fibers to reach the external granular layer (Yamada et al., 2000; Yamada and Watanabe, 2002). Studies on the *reeler* and *weaver* mutants has shown that SLC1A3 is down regulated in cerebellar astrocytes associated with Purkinje cells (Fukaya et al., 1999).

Reelin is also important for GFAP positive glial cell differentiation, process extension and orientation (Forster et al., 2006). In comparison to wild type mice, *reeler* mutant mice have unusual, numerous and heavily stained astrocytes with GFAP (Benjelloun-Touimi et al., 1985). Interestingly, in *scrambler* mutant mice, the cerebellum is small with no foliation, with GCs placed normally but their number reduced, and Purkinje cell numbers decreased and placed ectopically (Goldowitz et al., 1997). Goldowitz et al. (1997) showed that the effects of Reelin on Purkinje cells could also be mediated indirectly by Bergmann glia. Although these studies indicate the role of the Reelin pathway in neuronal migration and Bergmann glia cell development, there is no evidence that Purkinje cells are using somal transduction or glial guided migration (Schilling, 2018). Therefore, it is possible that Purkinje cells utilize a different mode of migration in which they disperse passively with the assistance of a pulling force from the Bergmann glia by regulating cell adhesion molecules to form a Purkinje cell monolayer (Figure 1). This should be examined in future studies.

### GABAergic Interneurons (Stellate/Basket and Golgi Cells)

Precursors of stellate and basket cells are generated within the VZ prenatally (when they express the paired homeobox gene, *Pax2*) and then migrate from the cerebellar plate to the developing white matter and postnatally (in mice) through the folial white matter while continuing to undergo cell division (Wefers et al., 2018). Thereafter, they migrate radially towards the molecular layer to accumulate at the inner border of the external granular layer and then migrate tangentially before settling at their final position within the molecular layer. A new study published by Wefers et al. (2018) document that the movement of cerebellar interneurons, basket cells and stellate cells, are highly directed and rerouted to the molecular layer during their transit through the nascent cerebellar cortex. They also showed that both the speed and directional persistence of basket cells and stellate cells are larger in the nascent GC layer than in the molecular layer (Wefers et al., 2018).

Golgi cell precursors are GC layer inhibitory interneurons and are born prenatally within the VZ. From the cerebellar plate these cells migrate to developing white matter while continuing proliferation during the migration until around P4 (Zhang and Goldman, 1996; Weisheit et al., 2006). During the perinatal development, Golgi cell precursors continue the migration through the developing folial white matter and terminate migration postnatally within the developing granular layer (Maricich and Herrup, 1999). In addition, a subset of Golgi cells are derived from the external germinal zone (Chung et al., 2011). These cells migrate within the white matter and become postmitotic postnatally and then migrate to position within the granular layer (Yamanaka et al., 2004; Wefers et al., 2018).

Although the mode and direction of migration of the GABAergic interneurons of the cerebellar cortex is a complex process and not entirely clear from origin to final position. However, based on the evidence the migration within the cerebellar cortex could be in the random walk mode.

### Granule Cell Origin, Migration and Final Destination

The GCPs that originate in the RL (Atoh1 expressing progenitors) migrate tangentially through a subpial stream pathway, and over the cortical surface to form the external germinal zone, similar to the rostral migratory stream from the ganglionic eminences to the olfactory bulb (Komuro and Yacubova, 2003; Stenman et al., 2003; Machold and Fishell, 2005). Simultaneously, GCPs co-express *Pax6, Meis1, Zic1/2* and *Barhl1* while post mitotic (mature) GCs do not express *Atoh1* (Stoykova and Gruss, 1994; Ackerman et al., 1997; Miyata et al., 1999; Morales and Hatten, 2006). Although the existence of a substrate or scaffold in tangential migration has not been confirmed, it is possible that the pial meninges have this role as these processes are present underneath the pial surface (Komuro and Yacubova, 2003). In addition, the external germinal zone is unique among proliferative germinal zones of the CNS as it is adjacent to the pial surface rather than the ventricular surface. The cells in this layer are highly proliferative, generating an enormous number of granule cell progeny, thereby greatly increasing the thickness of the external germinal zone. In mice at E12.5 to E17, GCPs are born and migrate to establish the external germinal zone (postmitotic GCs typically sojourn for 1–2 days within the lower layers of the external germinal zone) and give rise to GCs during the first two postnatal weeks (Figure 1; Komuro et al., 2001; Wang and Zoghbi, 2001). In humans, the external germinal zone is distinguished as a distinct layer between 10 weeks gestation to 2 month postnatally and will disappear by about year one and a half (Marzban et al., 2015). GCs initially follow a tangential migration and after proliferation in the external germinal zone, the cells migrate radially. The GCs situated in the inner layers of the external germinal zone start redial migration along Bergmann glial fiber to form the granular layer while expressing *NeuroD1* (an early marker of the differentiated GCs). The expression of *Unc5h3* and *Pax6* continues throughout the life span (Komuro and Yacubova, 2003). The granule cells also change from a round cell to a more horizontal-oriented shape as they begin to extend axons tangential to the cortical surface. The CXCR4, a G-protein-coupled chemokine receptor, is broadly expressed in cells of the CNS and can mediate migration in response to its ligand, stromal derived factor 1 (SDF-1; also known as chemokine ligand 12, CXCL12). The CXCR4/CXCL12 signaling pathway is involved in the migration of GCPs in the rostral migratory stream from the RL. The alterations in this pathway result in the movement of GCPs toward deeper positions away from the meninges, i.e., the inward radial migration, to form the granular layer (Leto et al., 2016). In the GC migration pathway, Sema6A functions in the switch from tangential migration in the external germinal zone to radial migration along Bergmann glia (Leto et al., 2016).

These postmitotic GCs migrate radially inward from the external germinal zone and pass by the developing Purkinje cell layer, to generate the granular layer. The cells migrate along the processes of the Bergmann glia, which is only present in the cortex of the cerebellum (Figure 1). Electron microscopic studies have detected Bergmann fibers in the external germinal zone by E15.5 in mice, and by 9 weeks gestation in humans (Choi and Lapham, 1980). The radial migration of the cerebellar granule neurons depends on actomyosin of the leading-process which coordinates organelle positioning and adhesion receptor dynamics (Ballif et al., 2004). During cerebellar development, DCX is strongly expressed by migratory GCs (as occurs in Purkinje cells) to mediate coupling of the nucleus to the centrosome (Gleeson and Walsh, 2000; Deutsch et al., 2010). Shh which is expressed by Purkinje cells plays a key role in GC proliferation, and may also provide a stop signal for GC proliferation and the beginning of the terminal differentiation as these cells migrate toward the source of Shh in Purkinje cell layer (Lewis et al., 2004). On the other hand, it is possible that the migration and maturation of GCs is not associated with Shh pathway signaling, but rather the subsequent loss of precursor cells from the external germinal zone limits the period over which postmitotic GCPs are generated (Lewis et al., 2004). The cellular and molecular mechanisms of cerebellar GC migration is reviewed by Yacubova and Komuro (2003).

### Cerebellar Nuclei Neurons Origin, Migration and Final Destination

During the early stages of the cerebellar development *Atoh1*-expressing neural progenitors, which arise from the RL at around E9.5–12.5, give rise to the excitatory (glutamatergic) CN projection neurons (Manto et al., 2013; Marzban et al., 2015). The CNN precursors migrate tangentially from the RL through the rostral migratory stream to midway of the cerebellar primordium and then change direction toward the NTZ, a transient cell mass that is subsequently partitioned and organized to form the medial, interposed and lateral CN (Fink et al., 2006; Kurosaka and Kashina, 2008). During development of the glutamatergic CNNs, transcription factors *Pax6*, *Tbr2*, *Tbr1 and Lmx1a* are expressed sequentially within the neurons of the RL and the NTZ (Fink et al., 2006). It has been shown that *Tbr1* knockout mice have a similar number of CNNs, but the boundaries are not properly delineated (Fink et al., 2006). Neuroblasts that express Pax6/Reelin move radially to become Tbr2 positive cells (Fink et al., 2006). The formation of the dentate nucleus (the largest and most lateral cerebellar nucleus) begins by stage 20 (52 days in humans) when precursors (interneurons INs and projection neurons) migrate radially from the VZ and rostromedially from the RL (Marzban et al., 2015). In mice, projection neurons of CN originate from the RL and unipolar neuroblasts migrate in the subpial stream toward the NTZ under the guidance of both diffusible Netrin (a family of laminin-related secreted proteins) and Slit (an extracellular matrix protein; Fink et al., 2006; Guerrini and Parrini, 2010; Matsuki et al., 2013; Qin et al., 2017). Interestingly, a study using quail-chick chimeras has shown that the rostromedial end of the cerebellar primordium originates from the alar plate of the caudal mesencephalon (Hallonet and Alvarado-Mallart, 1996). By mapping the Ptf1a promoter with a reporter, it was shown that the VZ only gives rise to inhibitory neurons of the CN. Thus the CNNs are assembled in a coordinated fashion through integration of PTF1a^+^ and ATOH1^+^ lineages in local circuits that migrate from two different germinal zones (Leto et al., 2016). Nichols and Bruce (2006) hypothesized that the mesencephalic neural crest is the source of several migratory streams and it is the dorsal intermediate stream that gives rise to the neurons of the CN.

During brain development, in the majority of brain regions (including CN) the CXCR4/CXCL12 signaling pathway is the most important signaling pathway that regulates neuronal migration. This receptor first appears in immature neurons in the RL at E12.5 (Tissir et al., 2004) and the expression of this receptor continues in the RL-derived CN precursors during the rostral subpial migratory stream pathway to the NTZ. Their ligand CXCL12 is expressed simultaneously in the meninges overlying this migratory pathway. Similar to tangential migration of GCPs, this indicates the potential interaction of CXCR4- and CXCL12-expressing cells in the rostral migratory stream. This may facilitate the rostral migration of RL-derived neurons and also change the direction of the migration toward the NTZ. This occurs through the detachment of neurons in this region from the pial surface to descend toward deeper positions in the NTZ (Tissir et al., 2004).

Although there is some evidence for tangential migration of CNNs from the RL subpially to the midway of the cerebellar primordium, the mode of the migration and the substrate utilized during the change of the direction from the pial surface of the cerebellar primordium to the NTZ are not known. Furthermore, the mode of migration and the substrate pathway for the putative subset of mesencephalic derived cerebellar nuclear neurons are also not clear and need to be addressed.

### Unipolar Brush Cell Origin, Migration, Final Position

UBCs project directly to GCs and amplify vestibular inputs to the cerebellum. In mice, the UBCs are generated from E13.5 to the early neonatal period (P0.5; Marzban et al., 2015). These cells originate from the RL and migrate through the developing white matter before settling in the GC layer of the cerebellar cortex (Hevner et al., 2006). The translocation of the UBCs from white matter to GC layer occurs between P3 to P10 and these cells sojourn in white matter for a period of time (Englund et al., 2006). Loss of the neurofibromatosis type 1 (*Nf1*) gene leads to imbalance in generating the appropriate numbers of glial cells, GC/UBC fate-specification/differentiation and GC/UBC migration (Kim et al., 2014). Studies have also shown a role for DCX in the neurogenesis and migration of UBCs (Manohar et al., 2012; Paolone et al., 2014). Furthermore, Wnt1 glycoprotein expression in the upper RL and UBCs is related to molecular identity and cell migration in cerebellar development (Hagan and Zervas, 2012). A previous study by Englund et al. (2006), reported that Tbr2 positive UBCs migrated from RL explants directly into the developing white matter of adjacent cerebellar slices (Hevner et al., 2006).

## Animal Models in Neuronal Migration

The use of animal models is a powerful approach to understand both human disease and basic biology (Schofield et al., 2012). Several human developmental malformations have been attributed to defects in neuronal migration and have been confirmed in mouse models in which a gene mutation results in abnormal neuronal migration (Table 2).

**Table 2:** Mutant mice models with cerebellar neuronal migration defects.

Mouse model	Gene	Function	Defect	Reference

*Reeler* (like lissencephaly 2 or Norman-Robert syndrome in human)	*Reln*	Neuronal migration (Purkinje cells in cerebellum and pyramidal cells in cerebral cortex)	Ectopic Purkinje cell cluster (~80%), no foliation, cerebellar hypoplasia	Goffinet (1983), Yuasa et al. (1993) and Miyata et al. (2010)
*Scrambler*	*Dab1*	The Reelin-Dab1 signaling pathway involves in neuronal migration and also in lamination	Ectopic Purkinje cell cluster, no foliation, cerebellar hypoplasia	Chung et al. (2007, 2009b)
*VLDLR/ApoE2*	*VLDLR/ApoE2*	Reelin receptors involves in neuronal migration and also in lamination	Ectopic Purkinje cell cluster, no foliation, cerebellar hypoplasia	Reddy et al. (2011)
*Src/Fyn*	*Src/Fyn*	Downstream molecules of Reelin signaling pathway involves in neuronal migration and also in lamination	Ectopic Purkinje cell cluster, no foliation, cerebellar hypoplasia	Kuo et al. (2005)
*Straggerer*	*RORa*	RORa is a gene expressed only in Purkinje cells in the olivocerebellar circuit	Purkinje cells are small, ectopic, possess rudimentary dendritic arbors and their number is reduced by about 75%. All of the granule cells and 60% of the inferior olivary neurons die during the first postnatal month.	Hadj-Sahraoui et al. (1997)
*Pten*	*Pten*	Pten express in Bergmann glia (scaffold)	Ectopic Purkinje cells and Purkinje cell dendritic arborization	Yue et al. (2005)
*SmoA2*	*SmoA2*	Member of SHH pathway	Ectopic clusters of Purkinje cells with disorganized dendritic arbors and axonal collaterals	Armengol and Sotelo (1991) and Dey et al. (2012)
Naked Ataxic (*nax*)	*Acp2*	Lysosomal acid phosphatase 2	Excessive migration of Purkinje cells to the molecular layer, no Purkinje cell monolayer formation, cerebellar hypoplasia, reduced granule cells proliferation	Mannan et al. (2004); Bailey et al. (2013, 2014); Rahimi-Balaei et al. (2016, 2018) and unpublished data
*p35/Cdk5*	*p35/Cdk5*	Cyclin-dependent kinase 5 and its regulator, p35 involve in neuronal migration, proliferation and neurite outgrowth	Normal gross morphology, folia and lamination. Molecular layer with more cell density (GCs) and ectopic PCs in granular layer	Chae et al. (1997)
*Weaver*	*Girk2*	G protein-activated inward rectifier potassium channel 2	Beside PCs and GCs death, neuronal migration defect as a result of Bergman glia abnormality	Rakic and Sidman (1973)
*Rp58*	*Rp58*	GABAergic and Glutamatergic neuron development	Severe cerebellar hypoplasia and developmental failure of Purkinje cells, Bergmann glia and granule cells	Baubet et al. (2012)
*CXCR4*- and *SDF-1* deficient	*CXCR4*- and *SDF-1(*aka *CXCL12*)	The chemokine receptor 4 (CXCR4)	#x02013;chemokine ligand 12 signaling pathway involve in neuronal migration and proliferation	Abnormal cerebellum, ectopic PCs, irregular external germinal zone	Ma et al. (1998), Larouche and Hawkes (2006) and Huang et al. (2014)
*Astn* or *Pex2*	*Astn* or *Pex2*	Genes for glial-guided neuronal migration	Ectopic granule cells precursors, abnormal Purkinje cell dendrite development, and external germinal zone present until late childhood cerebellum	Faust (2003)
*BDNF*	*BDNF*	Purkinje cells provide BDNF and promote granule cells precursors to differentiate and migrate along Bergmann glia fibers	Defects in cerebellar patterning such as ectopic granule cells precursors	Borghesani et al. (2002)
*Rb/p107*	*Rb/p107*	Survival of granule cells	Purkinje cells are disarranged with dystrophic dendrites	Marino et al. (2003) and Sotelo and Dusart (2009)
*VPS18*	*VPS18*	Disrupting multiple vesicle transport pathways to lysosomes	Neurodegeneration and impaired neuronal migration	Peng et al. (2012) and Davies (2013)
*MDM2*	*MDM2*	Link between p53 and Shh signaling pathways in granular neuronal precursors	Reduced levels of MDM2 and increased levels of p53 have small cerebella with shortened folia, reminiscent of deficient Shh signaling	Malek et al. (2011) and Gil-Sanz et al. (2013)
*Tbr1*	*Tbr1*	Cerebellar nuclei migration	Defect in medial cerebellar nuclei plus lateral and interpose	Fink et al. (2006)

In *reeler* knockout mice, the first manifestation of PCP malformation is at E14.5 and is prominent during cerebellar foliation at around E17.5 (Goffinet, 1983; Yuasa et al., 1993; Hadj-Sahraoui et al., 1997). This is mutation produces severe disorders in cellular migration throughout the brain and in the cerebellum it results in defects in Purkinje cell positioning, decreased proliferation and migration of GCs, and abnormality in foliation (Trommsdorff et al., 1999). The reduction and migratory defects observed in GCs could be due to Shh insufficiency caused by ectopically located Purkinje cells, which is far away from external germinal zone. The number of UBCs is also decreased and the cells are not positioned correctly (Trommsdorff et al., 1999). Interestingly, while there is no abnormality in the development of the NTZ in *reeler* knockout mice, the CN are significantly affected. The organization within the CN is especially disrupted in the lateral and medial CN. However, it has also been reported that alterations in the expression of the genes encoding the proteins in the Reelin signaling pathway do not change the morphology of the CN in mice. These components of Reelin pathway are cell surface receptor molecules VDLR/ApoER2, and intracellular signaling molecules Dab1, and tyrosine kinases Src and Fyn. In *Dab-1* mutant, *scrambler* mice, neurons show increased adhesion to radial glia which prevents them from reaching their final destination. These mice are ataxic and exhibit several neuroanatomical defects reminiscent of *reeler* mice. These findings indicate that abnormalities in the regulation of Reelin pathway result in cerebellar cortex anomalies which also result in defects in the development of CN (Fatemi, 2005).

In mice with a point mutation in the Lysosomal Acid phosphatase 2 (Acp2) gene, the result is a cerebellar defect with excessive migration of Purkinje cells to the molecular layer (Bailey et al., 2013, 2014; Rahimi-Balaei et al., 2016, 2018). We have recently investigated the role of Reelin-Dab1 signaling and its relationship to Erk1/2 (a member of mitogen activated kinases family) during Purkinje cell monolayer formation in the *Acp2* mutant cerebellum. Our findings indicate that down regulation of Reelin together with up regulation of phospho-Dab1 leads to the excessive and incorrect Purkinje cell migration in the *Acp2* mutant mice (under revision; Ashtari, 2017). In addition, it has been shown that the vacuole protein sorting 18 (VPS18), a core protein in intracellular vesicle transport, is involved in neuronal survival and CNS development. Genetic deletion of VPS18 leads to neurodegeneration and impaired neuronal migration as a result of disruption of multiple vesicle transport pathways that produce lysosomes. These findings indicate the importance of lysosomes in neuronal migration (Peng et al., 2012).

It is known that mouse double minute 2 homolog (MDM2), also known as E3 ubiquitin-protein ligase is a link between p53 and Shh. Using a p53 inhibitor it was shown that MDM2 is part of a signaling pathway in the development of GCs. It was reported that mice with reduced levels of MDM2 and increased levels of p53 have small cerebella with shortened folia, and Purkinje cells remained multi-layered and disorganized and exhibit stunted dendritic arborizations (Malek et al., 2011).

It has been shown that the lack of either *Astn* or *Pex2* (genes for glial-guided neuronal migration) produces a slowed migration pattern of GCPs which results in the formation of ectopic GCPs, abnormal Purkinje cell dendrite development, and the external germinal zone remains present until late childhood (Faust, 2003). Purkinje cells are an important source of brain-derived neurotrophic factor (BDNF) which promotes GCPs to differentiate and migrate along Bergmann glial fibers. Indeed, mice lacking BDNF have defects in cerebellar patterning such as ectopic GCPs (Borghesani et al., 2002). Finally, the deletion of CXCR4 leads to the premature migration of GCPs away from the proliferative zone of the external germinal zone, and small numbers of GCPs are found ectopically outside of the external germinal zone (Ma et al., 1998). Mouse models of fetal alcohol spectrum disorders and Minamata disease (a result of exposure to alcohol or methyl mercury during development) are also associated with deficits in GC migration related to interruption of a Ca^2+^/cyclic nucleotide signaling pathway (Komuro et al., 2015).

Another study by Baubet et al. (2012) has shown that the ablation of 58 KDa repressor protein (Rp58) results in severe cerebellar hypoplasia and failure of Purkinje cells, Bergmann glia and granule cells to develop properly which leads to a delay in the formation of the primary fissure, number of folia and defective lamination of the cerebellar cortex. Marino et al. (2003) have investigated the role of Rb/p107 in the development of the cerebellum; and have shown that it is involved in the survival of granule cells. In Rb-deficient and Rb/p107 double mutants, Purkinje cells are disarranged with dystrophic dendrites. In Phosphatase and tensin homolog (*Pten*) mutant mice ectopic Purkinje cells are present (Yue et al., 2005). Similarly in mice with a genetic deletion of either smoothened (*SmoA2*, member of Shh pathway) or *Cxcr4*, ectopic clusters of Purkinje cells are present with disorganized dendritic arbors and axonal collaterals (Dey et al., 2012; Huang et al., 2014).

Early postnatal mice with a mutation in *Tbr1*, have abnormal morphogenesis of the medial CN suggesting that migration defects are associated with malformation of this region of cerebellum. It is important to note that *Tbr1* mutation is associated with the irregular formation of medial CN, as well as irregular formation of interposed and lateral nuclei. Interestingly, although there are some histologic malformations following *Tbr1* mutation, these changes are not correlated with the neuronal loss, cell death, or axonal abnormalities (Fink et al., 2006).

## Neuronal Migration Disorders

Neuronal migration and positioning are critical processes during CNS development and circuitry formation, and defects in neuronal migration can lead to devastating brain diseases (Manto et al., 2013). It is well known that malfunctioning of the migratory process causes neuronal migration disorders (NMDs). NMDs are a heterogeneous group of birth defects with the same etiopathological mechanisms caused by the abnormal migration of neurons in the developing brain. This can result in neurological disorders with clinical manifestations including schizophrenia, autism, ataxia and epilepsy (Gleeson and Walsh, 2000; Nadarajah et al., 2003; Deutsch et al., 2010; Guerrini and Parrini, 2010; Demkow and Ploski, 2015; Marzban et al., 2015; Qin et al., 2017). The role of the Reelin pathway in neuronal migration has been extensively studied and in humans homozygous mutations in the *RELN* gene are associated with ataxia, cognitive abnormalities and cerebellar hypoplasia. In this context it has been also shown that the abnormal migration of cortical neurons is associated with reduced number of cortical gyri (lissencephaly). These results suggest an important role for Reelin in neuronal migration during the development. It should be noted that decreased levels of *RELN* expression have severe negative effects on the development of the human brain and may result in psychiatric diseases. For instance, patients who suffered from schizophrenia had reduced levels of *RELN* expression in the inhibitory neurons of their cortical areas. Additionally, decreased expression of Reelin has been observed in patients with other mental diseases, such as autistic-like disorders, bipolar disorder and major depressive disorder. Together these results suggest that Reelin has an important role in neuronal migration and synapse formation and deficits in Reelin expression may contribute to the pathophysiology of these disorders (Fatemi, 2005).

## Conclusion

Neuronal migration is all about: where do neurons come from (origin), where do they go (neuronal migration pathway), and what are they going to become (differentiated neurons and positioning)? Given that different subsets of neurons may migrate long or short distances in different modes and directions before the positioning, there is no doubt that an accurate and precise regulation of neuronal migration is necessary in order to establish the appropriate neuronal architecture and perturbations during development can result in neuronal migration disorders. During the development of the brain, proliferative germinal zones have two important tasks which are: (1) to produce the right number of cells for the particular brain region (either too many or too few will result in abnormalities); and (2) to produce the right class of cells that need to migrate to the right position. The delineation of the regulation of these two tasks is a major goal of developmental neuroscience. In this review we have examined neuronal migration and its different modes with a focus on cerebellar cell types.

## Author Contributions

All authors listed have made a substantial, direct and intellectual contribution to the work, and approved it for publication.

## Conflict of Interest Statement

The authors declare that the research was conducted in the absence of any commercial or financial relationships that could be construed as a potential conflict of interest.

## Abbreviations

Acp2: lysosomal acid phosphatase 2
ApoER2: apolipoprotein E receptor 2
Astn: astrotactin 1
Atoh1: atonal homolog 1
BDNF: brain-derived neurotrophic factor
CAMs: cell adhesion molecules
CN: cerebellar nuclei
CNN: cerebellar nuclei neuron
CNS: central nervous system
CXCL12: chemokine ligand 12
CXCR4: chemokine receptor 4
Dab1: disabled-1
DCC: deleted in colorectal cancer
DCX: doublecortin
E: embryonic day
EAAT1: excitatory amino acid transporter
EGF: epidermal growth factor
ErbB4: Erb-B2 receptor tyrosine kinase 4
FoxP2: forkhead box protein P2
GABA: gamma amino butyric acid
GC: granule cell
GCP: granule cell precursor
GFAP: glial fibrillary acidic protein
GLASTs: glutamate receptors and transporters
HGF/SF: hepatocyte growth factor/scatter factor
i: isthmus
IN: interneuron
IZ: intermediate zone
KCC2: potassium-chloride co-transporter
LIS1: lissencephaly-1 homolog
Lmx1a: LIM homeobox transcription factor 1 alpha
m: mesencephalon
MAP: microtubule-associated protein
MDM2: mouse double minute 2 homolog
MZ: marginal zone
*Nf1*: neurofibromatosis type 1
NRG1: neuregulin-1
NTZ: nuclear transitory zone
P: postnatal day
Pax2: paired homeobox gene 2
Pax6: paired homeobox gene 6
PC: purkinje cell
PCC: purkinje cell cluster
PCP: purkinje cell plate
Pex2: peroxisomal biogenesis factor 2
Ptf1a: pancreas specific transcription factor 1a
RL: rhombic lip
Rp58: 58 KDa repressor protein
SDF-1: stromal derived factor 1
Sema3A: semaphorin 3a
Sema6A: semaphorin 6A
Shh: sonic hedgehog
SmoA2: smoothened A2
SVZ: subventricular zone
Tbr1: T-box, brain, 1
Tbr2: T-box, brain, 2
UBC: unipolar brush cells
uPAR: urokinase-type plasminogen activator receptor
VLDLR: very-low-density lipoprotein receptor
VPS18: vacuole protein sorting 18
VZ: ventricular zone.
